# The Relationship Between Subjective Age, Health Status, and Advance Care Planning

**DOI:** 10.1093/geroni/igaa057.1356

**Published:** 2020-12-16

**Authors:** Ilana Engel, Tamara Baker

**Affiliations:** University of Kansas, Lawrence, Kansas, United States

## Abstract

Current data show that how old a person feels, or subjective age (SA), may be associated with improved well-being and functioning, less cognitive impairment, and longer life. Yet, the relationship between SA and advance care planning (ACP) has yet to be examined. This is all the more important when determining the role SA has in end-of-life decision-making. Using data from the Health and Retirement Study (Wave 13, 2016), this study aimed to examine whether SA is associated with having a living will (LW), having a LW with life-limiting care, and having assigned a durable power of attorney. The sample included 3,165 participants 51+ years of age (mean age = 65.67; SD = 11.79). Analyses were conducted assessing the predictive value identified social, behavioral, and health factors have for ACP. Results from binomial logistic regression analyses indicated that participants who endorsed feeling older than their chronological age were similarly likely to have engaged in ACP as those who felt younger. As demonstrated previously, older chronological age was significantly associated with higher utilization of ACP (ps < .05). Those with cancer and women were significantly more likely to have a LW (ps <.05). The present study did not find evidence to support a significant relationship between SA and ACP. Lower overall engagement with ACP and treatment for specific conditions, such as cancer, may be more influential in determining who utilizes ACP. Future research should explore how SA may serve as a protective factor and/or a psychological mechanism that influences engagement in ACP.

